# Optimal Design of Complementary Experiments for Parameter Estimation at Elevated Temperature of Food Processing

**DOI:** 10.3390/foods11172611

**Published:** 2022-08-28

**Authors:** Patnarin Benyathiar, Kirk D. Dolan, Dharmendra K. Mishra

**Affiliations:** 1 Department of Food Technology, Mahidol University, Kanchanaburi Campus, 199 Sangkraburi Road, Sai Yok, Kanchanaburi 71150, Thailand; 2 Department of Food Science and Human Nutrition, Michigan State University, 135 Trout Food Science Building, East Lansing, MI 48824, USA; 3 Department of Food Science, Purdue University, 745 Agriculture Mall Dr, West Lafayette, IN 47907, USA

**Keywords:** optimal complementary experiments, inverse problems, thermal properties, sensor design, food processing

## Abstract

Simultaneous estimation of thermal properties can be challenging, especially when the parameters are temperature-dependent. Previous research has shown that by using a complementary experiment, temperature-dependent thermal conductivity can be estimated using a single experiment. The objective of this study was to optimize the complementary experiments that can facilitate the simultaneous estimation of temperature-dependent thermal conductivity and volumetric heat capacity. A theoretical study was conducted with two experiments in a single trial with the sample being kept in a cylindrical sample holder, which had a thin film heater in the center. The first part of the experiment was conducted by keeping the external surface temperature at 50 °C for 300 s and allowing the center temperature to equilibrate with the boundary temperature. Then, the second part of the experiment followed, where the thin film heater was supplied with electrical power to increase the center temperate to 140 °C. Several heating profiles were studied to maximize the information obtained from the complementary experiments, and the best one was the power profile with a sinusoidal function. All four parameters of sweet potato puree temperature-dependent thermal conductivity (0.509 to 0.629 W/mK at 25 °C and 140 °C, respectively) and volumetric heat capacity (3.617 × 10^6^ to 4.180 × 10^6^ J/m^3^K at 25 °C and 140 °C, respectively) were estimated with low standard errors.

## 1. Introduction

Numerical simulations have played an important role in the development and optimization of the food manufacturing process to minimize trials and related costs. In food processing, computer simulations of the temperature profile of a product to determine food safety and quality have been applied for single-phase and multiphase models [1,2,3,4,5,6]. The process simulation requires parameters for accurate results. Moreover, the accuracy of the parameters is critical for a robust simulation of the process. To obtain accurate parameters, inverse problems of heat and mass transfer models have been used as a reliable and robust tool. Several researchers have investigated the estimation of thermal properties in different types of food thermal processes. The parameter estimation procedure and sensitivity analysis were used for thermophysical properties in bakery products [7,8], drying potato [9], grape pomace [10], potato puree [11], and low-moisture foods [12,13]. The thermal diffusivity, which is thermal conductivity over volumetric heat capacity, of food was estimated by performing an experiment with a constant boundary temperature condition in a water bath or in a retort thermal process [12,14]. However, in order to estimate thermal conductivity and volumetric heat capacity separately, a constant boundary temperature will not be sufficient, and a heat flux boundary condition must be used [15,16]. The accuracy of process simulation can be further improved by utilizing temperature-dependent thermal properties [11].

The use of complementary experiments has not been explored much for the estimation of temperature-dependent thermal properties. Complementary experiments are a combination of multiple experiments in a single trial, which improves the sensitivity coefficients of the parameters. The idea is to maximize the information that can be gained from such experiments [17]. The complementary experiment can be conducted in a single experimental setup, and it provides savings in cost and labor while improving the results. The correlation between parameters can be minimized to improve the confidence region of estimated parameters [18]. McMasters et al. 2018 showed that a single analysis can be conducted from multiple experiments to estimate the thermal conductivity and volumetric heat capacity.

The Thermal Properties Cell (TPCell) is a device that was developed to measure temperature-dependent thermal properties of food materials, such as puree and low-moisture food, using sequential parameter estimation [11,15]. The parameter estimation using the sequential estimation approach uses the Gauss-minimization method and provides more insight into the estimated parameters [19]. The special feature of TPCell is that it can estimate not only temperature-dependent thermal conductivity, but also the volumetric heat capacity. Due to the unique design of the heating element, it provides rapid heating up to 140 °C in less than a minute with the precise evaluation of temperature-dependent parameters [15]. A complementary design was used for the estimation of temperature-dependent thermal parameters, and the authors were able to simultaneously estimate the volumetric heat capacity along with the temperature-dependent thermal conductivity [20]. With regard to the previous study [20], the first part of the complementary experiment was a constant boundary temperature up to 50 °C, and the second part of the complementary experiment was to provide heat to the center of the sample through a cartridge heater up to 140 °C. However, it was not possible to estimate the temperature-dependent volumetric heat capacity due to the low sensitivity coefficient based on the temperature profile. Design improvements are hence needed to ensure that the temperature-dependent volumetric heat capacity can be estimated with good accuracy. Hence, the novel approach of a complementary experimental design was implemented in this study. The objective of this study was to develop an optimal complementary experimental design to estimate the thermal properties, including temperature-dependent thermal conductivity and temperature-dependent volumetric heat capacity, in the temperature range of 20–140 °C.

## 2. Materials and Methods

### 2.1. Sensor Design

**Figure 1:**
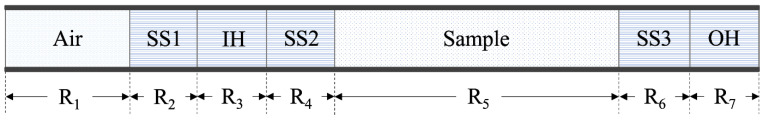
TPCell Schematic of 1D axisymmetric model. Air—air inside the hollow steel cylinder, SS1—thickness of hollow stainless-steel cylinder, IH—inner heater, SS2—thickness of stainless-steel sample holder, Sample—annular space for sample, SS3—thickness of sample holder, OH—outer heater, R—radial distance from the center of the steel cylinder, with different dimensions: R1 = 2.48 mm, R2 = 2.98 mm, R3 = 3.37 mm, R4 = 3.87 mm, R5 = 9.32 mm, R6 = 9.83 mm, R7 = 10.21 mm.

The TPCell was created as a benchtop device to analyze the thermal properties of food products. It consists of four main components: (1) Two thin film heaters, (2) a stainless-steel base, (3) a stainless-steel sample holder, and (4) sample closure [20]. The performance of a fast heating and cooling rate can be obtained by the design of the heater on the stainless-steel base and copper coil around the outer sleeve, as illustrated in Figure 1. Commercially available sweet potato puree for baby food (Gerber^®^ 2nd foods brand, Florham Park, NJ, USA) was chosen as a model food sample for simulations in this study. The sweet potato puree’s moisture content was 84.1%.

### 2.2. Mathematical Model

The transient heat conduction in a hollow cylinder presents the system of TPCell with the following equations:

(1) $$\frac{1}{r}\frac{\partial }{\partial r}\left[k_{a}r\frac{\partial T}{\partial r}\right]=C_{a}\frac{\partial T}{\partial t}\;\mathrm{for}\;\;0\;<r\leq R_{1},\;t>0,$$

where *k_a_* is the thermal conductivity and *C_a_* is the volumetric heat capacity of air, and R_1_ is the inner radius of the hollow cylinder

(2) $$\frac{1}{r}\frac{\partial }{\partial r}\left[k_{s}r\frac{\partial T}{\partial r}\right]=C_{s}\frac{\partial T}{\partial t}{\;\mathrm{for}\;R}_{1}<r\leq R_{2},\;R_{3}\;<r\leq R_{4}{,\;R}_{5}<r\leq R_{6},\;t>0,$$

where *k_s_* is the thermal conductivity and *C_s_* is the volumetric heat capacity of stainless steel. R_1_ and R_2_ are the inner and outer radii of the hollow cylinder, respectively. R_3_ and R_4_ are the inner and outer radii of the sample holder’s inside wall, respectively. R_5_ and R_6_ are the inner and outer radii of the sample holder’s outside wall, respectively.

(3) $$\frac{1}{r}\frac{\partial }{\partial r}\left[k_{h}r\frac{\partial T}{\partial r}\right]+g_{0}\left(t\right)=C_{h}\frac{\partial T}{\partial t}{\;\mathrm{for}\;R}_{2}<r\leq R_{3},\;t>0,$$

where *k_h_* is the thermal conductivity and *C_h_* is the volumetric heat capacity of the thin film heater. R_2_ and R_3_ are the inner and outer radii of the heater, respectively. *g*_0_ is the heat generation term.

(4) $$\frac{1}{r}\frac{\partial }{\partial r}\left[k\left(T\right)r\frac{\partial T}{\partial r}\right]=C\left(T\right)\frac{\partial T}{\partial t}{\;\mathrm{for}\;R}_{4}<r\leq R_{5},\;t>0,$$

where *k*(*T*) is the temperature-dependent thermal conductivity and *C*(*T*) is the temperature-dependent volumetric heat capacity of the sample. R_4_ and R_5_ are the inner and outer radii of the sample annular space, respectively. The initial temperature is calculated by Equation (5):

(5) $$T\left(r,0\right)=T_{0},$$

The boundary condition at R_6_ is given by Equation (6):

(6) $$T\left(R_{6},t\right)=T_{Boundary}\;\left(t\right),$$

where *T_Boundary_* (*t*) is the temperature at the boundary R_6_.

The thermal conductivity and volumetric heat were analyzed using a linear relationship with temperature. The finite element method and COMSOL Multiphysics 6.0 (Burlington, MA, USA) were used to assess the 1D model.

### 2.3. Complementary Design of Experiments

The design of the complementary experiment was based on the power profiles of the individual heater and temperature measurements at two places as described in Figure 1. The non-complementary experiment was a simple pulse of power at the center of the sample. However, with this experiment, it was not sufficient to simultaneously estimate the temperature-dependent thermal conductivity and volumetric heat capacity [15]. Later, the experiment was modified to include two experiments using a complementary design (CD1) with a single inverse analysis [20]. In the first part of the complementary design, the outer heater was turned on and maintained at 50 °C and the temperature at the center of the sample (*T_Center_*) started at room temperature and then continuously increased to 50 °C by the heat flux of the outer heater. After *T_Center_* reached 50 °C, the inner heater generated 12 W of power for a duration of 120 s. This design enabled the estimation of *k*_1_, *k*_2_, and *C*_1_, but not *C*_2_.

In this study, optimal design criteria were used to access several complementary experimental designs that would enable the simultaneous estimation of *k*_1_, *k*_2_, and *C*_1_, and *C*_2_. The second complementary design (CD2) was designed to keep the center at a constant temperature once it reached 140 °C by tuning the inner heater power with a PID loop. The third complementary design (CD3) was chosen where the boundary temperature was modulated using a sine wave with a frequency of 0.05 hertz and an amplitude of 10. The center temperature was then increased to 140 °C by turning on the inner heater. For the fourth complementary design (CD4), the first part was the same as CD2, but the center heater was modulated with a sine wave of 0.05 hertz and an amplitude of 2. The CD4 experiment was terminated as soon as the center temperature reached 140 °C.

### 2.4. Optimal Experimental Design

To determine the optimal experimental design, the sensitivity coefficient of a parameter is first calculated by taking the first derivative of the dependent variable with respect to the parameter. The mathematical description is given in Equation (7) [19]:

(7) $$X_{i}=\frac{dT}{di},$$

where *X_i_* is the sensitivity coefficient for parameter *i*, and *T* is the dependent variable, which is the temperature in this case. To obtain the scaled sensitivity coefficient (SSC), the sensitivity coefficient is multiplied by the value of the parameter as shown in the following equation.

(8) $${\hat{X}}_{i}=i\frac{dT}{di},$$

The SSC needs to be large compared to the response variable in order to have a precise estimation of the parameter with low standard errors and smaller confidence intervals. Furthermore, when there are multiple parameters to be estimated, one SSC cannot be correlated with another parameter. A high correlation leads to uncertainty in parameters, and they cannot be estimated simultaneously. In the case of high correlation, the experiment must be modified, or additional experiments, such as a complementary experiment, must be added for the simultaneous estimation of the parameters.

Finding optimal experiments is one of the fundamental types of parameter estimation problems [19]. For the complementary design, it is essential to find which experiment could perform best for the estimation of the desired parameters. The criterion for optimal design is based on the minimization of the hyper-volume of the confidence region. For the evaluation of two or more parameters, an optimal experiment design requires the greatest determinant value of the sensitivity matrix. Mathematically,

(9) $$\Delta =\max\left|X^{T}X\right|,$$

where Δ is the optimal value and *X* is the sensitivity [*n* × *p*] matrix, with *n* measurements and *p* parameters. The optimality criterion can be described as:

(10) $$\max\;\Delta^{n}=\max\frac{\Delta }{n^{p}},$$

For four parameters (*p* = 4), with *n* measurements, the equation changes to

(11) $$\;\Delta^{n}=\frac{\Delta }{n^{4}},$$

The Δ value as a function of time was analyzed and normalized with the maximum power supply.

### 2.5. Inverse Problem

The use of a high temperature is common for thermal food processing and the shelf life of the product. The thermal properties, including specific heat requirements and thermal conductivity, are typically difficult to determine at high temperatures. The forward problem is used to calculate the predicted temperature using the initial guess values of the unknown parameters. The inverse problem can then be used to estimate parameters of interest from a given experimental temperature profile. The TPCell model was defined by Equations (1)–(6) using a combination of numerical finite element solutions in COMSOL and MATLAB^®^ R2022a (Natick, MA, USA). The inverse solution is sensitive to the errors of the measurement; hence, the accuracy of measurement is important for the accurate estimation of the parameters. The center temperature of the sample was obtained by embedding a thermocouple at the center of the inner heater. The initial temperature of the sample was specified as the equilibrated temperature of the TPCell at the start of the experiment (25 °C).

The sequential estimation of the parameter algorithm was used following Mishra et al. (2016) [15]. Sequential estimation was achieved by using the matrix inversion and Gauss minimization function, which can be explained by

(12) $$S={\left[Y-\hat{Y}\left(\beta \right)\right]}^{T^{\prime }}W\left[Y-\hat{Y}\left(\beta \right)\right]+{\left[\mu -\beta \right]}^{T^{\prime }}\dot{U}\left[\mu -\beta \right],$$

where *S* is the Gauss minimization function, *Y* is the experimental response variable, $\hat{Y}$ is the predicted response, β is the parameter, $T^{\prime }$ is the transpose of the matrix, *W* is the inverse of the covariance matrix of errors, and *μ* is the prior information of the parameter.

The extremum of the minimization function can be explained by differentiating the function with the parameter as represented below,

(13) $$\nabla_{\beta }S=-2{\left[\nabla_{\beta }\hat{Y}\left(\beta \right)\right]}^{T}W\left[Y-\hat{Y}\left(\beta \right)\right]-2\left[I\right]\dot{U}\left[\mu -\beta \right],$$

where *I* is the identity matrix. The steps of the inverse algorithm are given by Equations (14)–(19).

(14) $$A_{i+1}=P_{i}X_{i+1}^{T},$$

(15) $$\Delta_{i+1}=\emptyset_{i+1}+X_{i+1}A_{i+1},$$

(16) $$\;\;\;K_{i+1}=A_{i+1}\Delta_{i+1}^{-1},$$

(17) $$e_{i+1}=Y_{i+1}-{\hat{Y}}_{i+1},$$

(18) $$b_{i+1}^{*}=b_{i}^{*}+K_{i+1}\left[e_{i+1}-X_{i+1}\left(b_{i}^{*}-b\right)\right],$$

(19) $$P_{i+1}=P_{i}-K_{i+1}X_{i+1}P_{i},$$

The termination criterion for the parameter can be provided as

(20) $$\left[\frac{b_{j}^{k+1}-b_{j}^{k}}{\left|b_{j}^{k}\right|+\delta_{1}}\right]<\delta ,$$

where *X* is the sensitivity matrix, *P* is the covariance vector matrix of parameters, e is the error vector, i and j are the index of iteration, and *b* is the parameter index. To apply the inverse problem to estimate parameters in this study, several statistical assumptions were considered: (1) *Y* = η(*X*,*b*) + **e** where *Y* is the additive measurement error and η (regression function) does not contain any random errors; (2) zero mean of measurements, which can be verified with the residual analysis of the estimated parameters and predicted temperature, (3) constant variance of errors, (4) uncorrelated errors, and (5) the error has a normal distribution.

## 3. Results and Discussion

### 3.1. Complementary Design (CD1)

**Figure 2:**
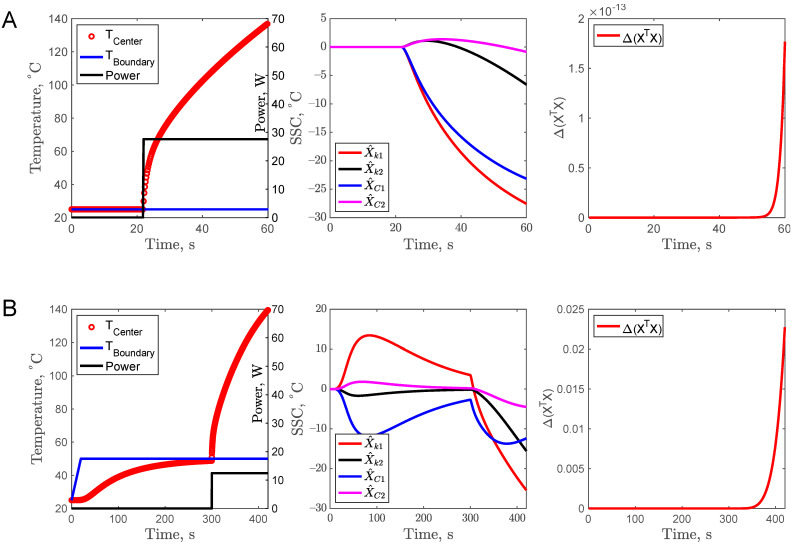
Non-complementary experiment (**A**), and complementary experimental design (**B**), temperature profile with power input (**left**), scaled sensitivity coefficients (**center**), and criteria for optimal design (**right**).

For all the experimental trials, the maximum temperature at the center of the sample was kept below 140 °C. A non-complementary experiment is presented in Figure 2A. The center temperature of the sample was increased by turning on the center heater at 20 s, and as a result, the center temperature attained 140 °C at the end of 60 s. It is worth noting that the SSC of thermal conductivity ${\hat{X}}_{k1}$ and ${\hat{X}}_{k2}$ are large and uncorrelated. However, the SSC of the volumetric heat capacity ${\hat{X}}_{C1}$ and ${\hat{X}}_{C2}$ are relatively very small compared to the total temperature rise of 120 °C. In addition, the ${\hat{X}}_{C1}$ and ${\hat{X}}_{k1}$ are correlated. Due to this reason, the parameters *C*_1_ and *C*_2_ could not be estimated with this specific experimental setup. The complementary design CD1 consisted of two experiments as shown in Figure 2B. The first experiment was to keep the sample cup at a constant boundary temperature of 50 °C and wait for 300 s for the center sample temperature to equilibrate with the boundary temperature. The second part of the complementary design CD1 was the same as in Figure 2A, where the center heater power was turned on at 300 s. As shown in Figure 2B, for the first experiment, before 300 s, it is expected that the ${\hat{X}}_{k1}$ is correlated with ${\hat{X}}_{C1}$, and ${\hat{X}}_{k2}\;$ is correlated with ${\hat{X}}_{C2}$ due to the constant temperature boundary condition [21]. However, there is a remarkable improvement in ${\hat{X}}_{C1}$ for the second experiment (after 300 s), but the same was not true for ${\hat{X}}_{C2}$. Hence, with the complementary design CD1, it is possible to simultaneously estimate parameters *k*_1_, *k*_2_, and *C*_1_ but not *C*_2_. The optimizing function Δ value also increased significantly to 2 × 10^−2^ as compared to 15 × 10^−14^ for the non-complementary design, proving that the CD1 design is better.

### 3.2. Constant Temperature Profile in Second Experiment of Complementary Design (CD2)

**Figure 3:**
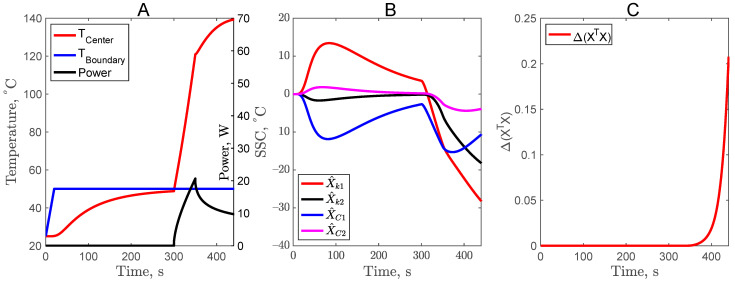
Center temperature profile with power input (**A**), scaled sensitivity coefficients (**B**), and criteria for optimal design (**C**) for the CD2 experimental design.

To further optimize the complementary design, a constant temperature at the center of the sample was considered for the second experiment (from 300 to 500 s) of complementary design CD2. As shown in Figure 3, to maintain a constant center temperature, a PID control was used for the heater power. With this approach, there was a slight improvement in ${\hat{X}}_{C2}$, but still it was not enough to estimate *C*_2_ with good accuracy. Nevertheless, the optimality criteria improved to 1.6 as compared to 2 × 10^−2^ for the CD1 design. Using CD2 design, the simultaneous estimation of *k*_1_, *k*_2_, and *C*_1_ will result in better RMSE and parameter standard errors.

### 3.3. Sinusoidal Boundary Temperature Profile in First Experiment of the Complementary Design (CD3)

**Figure 4:**
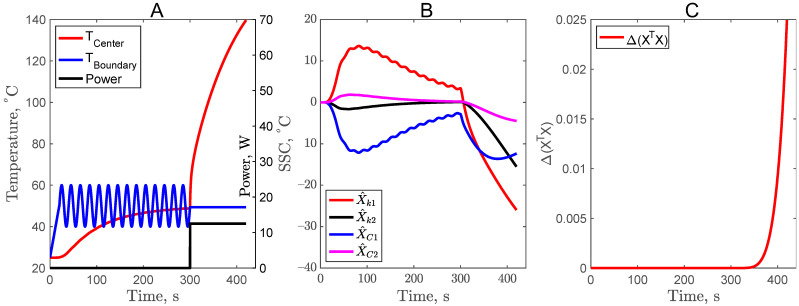
Center temperature profile with power input (**A**), scaled sensitivity coefficients (**B**), and criteria for optimal design (**C**) for the experiment with sinusoidal boundary temperature.

A sinusoidal function with a frequency of 0.05 and an amplitude of 10 was added to the boundary temperature profile to explore whether it would improve the optimality criteria. As observed in Figure 4, the sinusoidal profile did not improve the ${\hat{X}}_{C2}$ and it was merely 4% of the 120 °C total temperature. In the case of CD3, it would not be able to estimate *C*_2_ with good accuracy. In addition, the optimality criteria also remained the same as in CD1. So, the sinusoidal temperature profile at the sample boundary was discarded as an optimal design and was not further explored for inverse analysis and parameter estimation.

### 3.4. Sinusoidal Power Profile in Second Experiment of Complementary Design (CD4)

**Figure 5:**
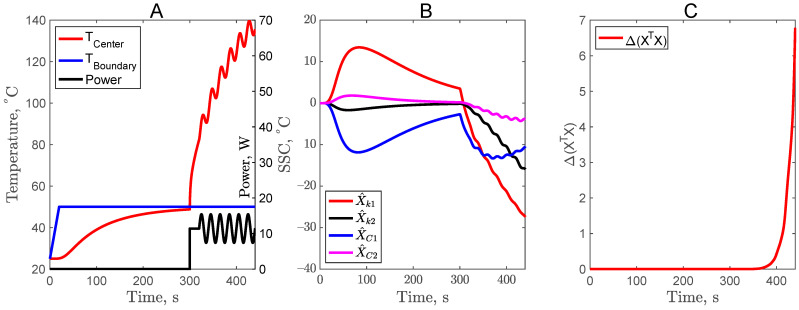
Center temperature profile with power input (**A**), scaled sensitivity coefficients (**B**), and criteria for optimal design (**C**) for sinusoidal center heater power profile.

Another approach of adding a sinusoidal function was implemented for the center heater power profile. The power was varied, starting at 300 s with a frequency of 0.05 hertz and an amplitude of 2 (Figure 5). When the center heater powder was turned on, the center temperature rise was also influenced by the sinusoidal profile. The power was turned off as the temperature reached 140 °C. The SSC, before the 300 s, remained the same as in CD1, CD2, and CD3. Due to the added sinusoidal function, the sensitivity after 300 s for ${\hat{X}}_{C2}$ increased slightly. However, the most significant impact was on the optimizing function Δ value, where it increased to 14 as compared to 2 × 10^−2^ for the CD3 design. This is a remarkable improvement and shows that CD4 can potentially be used for the simultaneous estimation of all four parameters, *k*_1_, *k*_2_, *C*_1_, and *C*_2_.

### 3.5. Impact of High Thermal Conductivity of Heater

**Figure 6:**
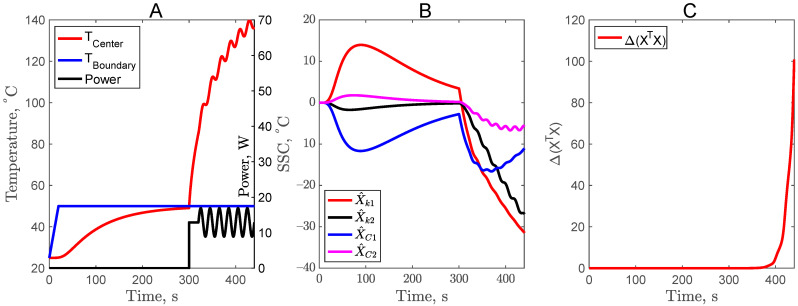
Center temperature profile with power input (**A**), scaled sensitivity coefficients (**B**), and criteria for optimal design (**C**) for the experiment with high thermal conductivity of heater.

The thermal conductivity of the heater is important in designing the device for measuring thermal properties. It was reported that the thermal conductivity of the thin film heater was 0.01 W/mK due to the various layers and internal contact resistance. If the construction of the heater can be improved with increased thermal conductivity, it might help the estimation of the sample’s thermal properties. Hence, to investigate the impact of the heater’s thermal conductivity, a higher value of 1.0 W/mK was chosen. All other aspects of the experiment CD4 were the same except for the thermal conductivity of the heater. The results are shown in Figure 6, and it is evident that there is improvement in ${\hat{X}}_{k1},$ ${\hat{X}}_{k2},$ ${\hat{X}}_{C1}$, and ${\hat{X}}_{C2}$. In addition, there is a 1428% increase (from 7 to 100) in the optimizing function Δ value as compared to the lower thermal conductivity case. Hence, the CD4 design along with the higher thermal conductivity of the heater is the optimal model and is further considered for the inverse problem analysis for the estimation of the four parameters (*k*_1_, *k*_2_, *C*_1_, and *C*_2_).

### 3.6. Parameter Estimation for the Optimal Complementary Design

**Figure 7:**
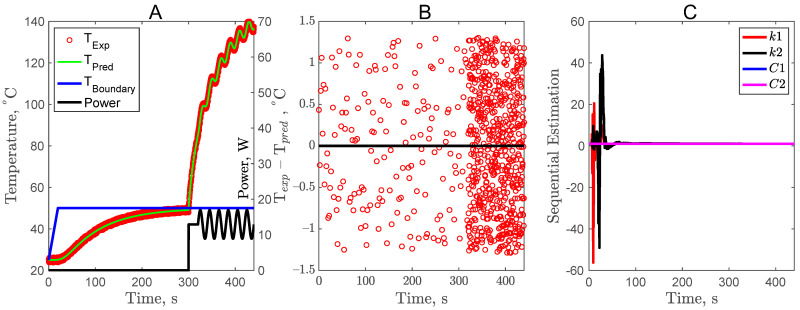
Inverse problem analysis and sequential parameter estimation of the optimal complementary design.

Based on the optimal complementary design, temperature data were generated to simulate an experiment. A random error (σ) was added to the simulated temperature profile using the forward problem solution. The inverse problem’s results are shown in Figure 7 to validate the results of simulation as presented in the forward problem and sensitivity coefficient analysis. As a sample food product, sweet potato was chosen for the thermal property estimation. As shown in Figure 7A, the sample was kept at a constant boundary temperature of 50 °C for 300 s until the center of the sample reached the boundary temperature. After the first part of the complementary experiment and sample equilibration, the center heater was turned on to supply a power of 12.9 W with an added sine wave at a frequency of 0.05 hertz and an amplitude of 4. The impact of adding a sine wave is visible in the second part of the complementary experiment, where the center sample temperature rises and follows the patterns of the sinusoidal power input and attains a maximum temperature of 140 °C. The predicted center temperature fits the first part of the complementary experiment well; however, as described above, it is only possible to estimate the thermal diffusivity and not the simultaneous estimation of thermal conductivity and volumetric heat capacity. It is the second part of the complementary experiment that allows for simultaneous estimation. The predicted center sample temperature also fits for the second part of the complementary experiment well. Residuals are presented in Figure 7B, and it can be observed that statistical assumptions of the zero mean and uncorrelated errors are true, which validates that the inverse problems are good. Based on the zero mean, uncorrelated errors, and uniformly scattered and normalized residuals, it can be concluded that the errors are additive in the temperature measurement. The multiplicative errors will not have constant variance and will be easily observed in Figure 7B.

To verify if the optimal complementary experiment is suitable for the simultaneous estimation of temperature-dependent thermal conductivity and volumetric heat capacity, a series of simulated experiments were carried out. As presented is Table 1, the random errors (σ = 0, 0.5 and 2) were added to the temperature profile. A sequential estimation approach was used for parameter estimation where the sample size is not fixed and, instead, it is sequentially added to the analysis and the outcome is determined. Sequential estimation is terminated when precision in the estimation is achieved. As shown in Figure 7C, all the normalized parameters achieved a constant value after 100 s of the experiment, suggesting the precise estimation of parameters. The estimated parameters for various random errors are presented in Table 1 along with the parameter standard error, RMSE, SSE, and confidence intervals. A higher experimental error greatly impacts the accuracy of estimated parameters. For example, a σ value of 2 resulted in higher RMSE, higher SSE, and larger confidence intervals. The most important fact to note here is that all four parameters (*k*_1_, *k*_2_, *C*_1_, and *C*_2_) can be accurately estimated with the optimal complementary experimental design (CD4).

The thermal conductivity for sweet potato puree presented in Table 1 was in the same range as the values reported by Mishra et al. [15], 0.518 to 0.548 W/mK at 25 °C and 0.572 to 0.585 W/mK at 140 °C. However, in their research, they did not estimate the volumetric heat capacity of sweet potato puree. Mehta et al. [20] attempted to design the complementary experiments and were able to simultaneously estimate the temperature-dependent thermal conductivity and a constant volumetric heat capacity. The estimated thermal conductivity was reported as 0.516 W/mK at 26 °C and 0.798 W/mK at 135 °C, whereas the estimated volumetric heat capacity was 3.520 × 10^6^ J/m^3^K at 26 °C. These values are in the same range reported in Table 1 Even in the study of Mehta et al., the temperature-dependent volumetric heat capacity was not estimated due to the non-optimal design of the complementary experiments. Hence, the novelty of this study was in the estimation of temperature-dependent volumetric heat capacity, which was found to be in the range of 3.617 × 10^6^ J/m^3^K at 25 °C to 4.180 × 10^6^ J/m^3^K at 140 °C.

## 4. Conclusions and Recommendations

An optimal complementary experimental design was presented for the estimation of the temperature-dependent thermal conductivity and volumetric heat capacity of sweet potato puree. Scaled sensitivity coefficients were used to discern if the parameter of interest can be estimated with accuracy. Several boundary conditions were used, and experimental designs were presented to investigate the impact of the complementary design. The results showed that the optimal design was the one where the first part of the experiment was a constant temperature boundary condition and the second part was the use of sinusoidal heat flux. Using such a complementary design, the estimation of two parameters of temperature-dependent thermal conductivity and two parameters of temperature-dependent volumetric heat capacity was made possible. Hence, the use of the complementary design was proven to be successful and has the potential for applications in many areas of food process engineering. One significant application will be in the processing of particulate aseptic food products where modeling using thermal parameters is necessary to design the thermal process. The future steps of this study will explore the manufacturing of a cylindrical, high-conductivity cartridge heater for manufacturing the device that can simultaneously estimate temperature-dependent thermal properties.

## Figures and Tables

**Table 1:** Impact of experimental error (σ) on the estimation of temperature-dependent thermal parameters.

σ	Symbol	Parameter	Standard Error	RMSE	SSE	LCI	UCI
0.00	*k*_1_ (W/mK)	0.510	0.001	0.000	0.000	0.510	0.510
	*k*_2_ (W/mK)	0.630	0.001	0.000	0.000	0.630	0.630
	*C*_1_ (J/m^3^K)	3.612 × 10^6^	0.003 × 10^6^	0.000	0.000	3.612 × 10^6^	3.612 × 10^6^
	*C*_2_ (J/m^3^K)	4.128 × 10^6^	0.027 × 10^6^	0.000	0.000	4.128 × 10^6^	4.128 × 10^6^
0.50	*k*_1_ (W/mK)	0.509	0.001	0.743	4190.500	0.506	0.512
	*k*_2_ (W/mK)	0.629	0.001	0.743	4190.500	0.625	0.632
	*C*_1_ (J/m^3^K)	3.617 × 10^6^	0.003 × 10^6^	0.743	4190.500	3.606 × 10^6^	3.629 × 10^6^
	*C*_2_ (J/m^3^K)	4.180 × 10^6^	0.027 × 10^6^	0.743	4190.500	4.128 × 10^6^	4.128 × 10^6^
2.00	*k*_1_ (W/mK)	0.496	0.001	2.978	67381.000	0.483	0.510
	*k*_2_ (W/mK)	0.638	0.001	2.978	67381.000	0.625	0.651
	*C*_1_ (J/m^3^K)	3.595 × 10^6^	0.003 × 10^6^	2.978	67381.000	3.551 × 10^6^	3.640 × 10^6^
	*C*_2_ (J/m^3^K)	4.504 × 10^6^	0.028 × 10^6^	2.978	67381.000	3.854 × 10^6^	5.146 × 10^6^

## Data Availability

The data presented in this study are available on request from the corresponding author.
